# The challenge of long waiting lists: how we implemented a GP referral system for non-urgent specialist' appointments at an Australian public hospital

**DOI:** 10.1186/1472-6963-10-303

**Published:** 2010-11-04

**Authors:** Lesley A Stainkey, Isaac A Seidl, Andrew J Johnson, Gladys E Tulloch, Tilley Pain

**Affiliations:** 1 Townsville General Practice Network, PO Box 7780, Garbutt BC, QLD 4814, Australia; 2 The Townsville Hospital, 100 Angus Smith Drive, Douglas, QLD 4811, Australia; 3 School of Medicine and Dentistry, James Cook University, Townsville, QLD 4811, Australia

## Abstract

**Our Problem:**

The length of wait lists to access specialist clinics in the public system is problematic for Queensland Health, general practitioners and patients. To address this issue at The Townsville Hospital, the GP Liaison Officer, GPs and hospital staff including specialists, collaborated to develop a process to review patients waiting longer than two years. GPs frequently send referrals to public hospital specialist clinics. Once received, referrals are triaged to Category A, B or C depending on clinical criteria resulting in appointment timeframes of 30, 90 or 365 days for each category, respectively. However, hospitals often fail to meet these targets, creating a long wait list. These wait listed patients are only likely to be seen if their condition deteriorates and an updated referral upgrades them to Category A.

**Process to Address the Problem:**

A letter sent to long wait patients offered two options 1) take no action if the appointment was no longer required or 2) visit their GP to update their referral on a clinic specific template if they felt the referral was still required. Local GPs were advised of the trial and provided education on the new template and minimum data required for specialist referrals.

**What Happened:**

In 2008, 872 letters were sent to long wait orthopaedic patients and 101 responded. All respondents were seen at specially arranged clinics. Of these, 16 patients required procedures and the others were discharged. In 2009 the process was conducted in the specialties of orthopaedics, ENT, neurosurgery, urology, and general surgery. Via this new process 6885 patients have been contacted, 633 patients have been seen by public hospital specialists at specially arranged clinics and 197 have required a procedure.

**Learnings:**

Since the start of this process in 2008, the wait time to access a specialist appointment has reduced from eight to two years. The process described here is achievable across a range of specialties, deliverable within the routine of the referral centre and identifies the small number of people on the long wait list in need of a procedure.

## Background to the problem

This article describes the trial of a service model to allow patients who have been wait listed for 2 years or more, as a non-urgent referral, to be seen at a public hospital specialist outpatient clinic. To access outpatient specialist clinics, patients must obtain a referral from a GP which is sent to the hospital where the referral is triaged prior to delegating appointments. The triage system prioritises referrals depending on clinical criteria with the most urgent assigned Category 1 and least urgent, Category 3. Queensland Health recommended timeframes for patients to be seen from date of receipt of the referral are 30, 90 or 365 days for Category 1, 2 or 3, respectively. However, for various reasons, The Townsville Hospital often does not have the capacity to meet these criteria. This results in a wait list of patients from Category 2, but mostly from Category 3, for which wait times extend beyond two years. These 'long wait' patients are unlikely to be seen unless their condition deteriorates and an updated referral upgrades them to a Category 1. A review of the process was warranted because of the potential that a small number of patients on the list may need a procedure. Also, a review could address the administrative difficulties and risk management issues associated with referrals that were inadequate and out of date.

The Townsville Hospital gained negative attention as having the second longest list of patients waiting for specialist appointments in the State. To address this issue, The Townsville Hospital collaborated with the GP Liaison Officer from the local Division of GPs (Townsville GP Network) to ensure cooperation from both primary care and hospital staff. The overall process included two strategies: 1) to provide access to appointments for patients who have been on the wait list for longer than two years by updating their referral using a specifically designed referral template, and 2) to streamline the process of new referrals coming into the system. The second strategy was considered necessary to ensure the number of new referrals entering the system does not overwhelm the hospital's capacity and develop into another long wait list. This communication describes the first strategy: the process to target long wait referrals where 'long wait' was defined as two years or older.

### What We Learnt from the Literature

Long wait times to access specialist outpatient consultations and associated procedures are endemic in public hospitals in Australia[1] and overseas [2-4]. Numerous detrimental factors come into play when wait lists become onerously long, including additional administrative support[5] and increased mortality and morbidity rates [6]. Wait lists are inflated by patients that have not been investigated thoroughly prior to referral reducing capacity for accurate triaging [7]. To improve clinical management of patients on waiting lists, innovative models of care have been widely adopted including the Orthopaedic Physiotherapy Screening Clinic, nurse practitioner first contact clinics and a remote rheumatology outpatient clinic [5]. The process we are developing includes patients, GPs and specialists. Once GPs are provided with information about waiting times, their willingness to change their referral practice rises [8]. Patients increasingly want greater involvement in making decisions regarding their care [9] and as it is generally accepted for patients to initiate care. Therefore, it should also be accepted that patients can elect to withdraw from care.

## How the process was implemented

### Who was involved

The overall coordinator for the process was the Townsville GP Network's GP Liaison Officer. Key hospital staff included the Nurse Unit Manager of Surgical Specialist Clinics, and the Executive Director of Medical Services. The referral templates were developed in collaboration between The Townsville Hospital specialists from the relevant specialty and the GP Clinical Reference Group from Townsville GP Network. The process was advertised and marketed extensively throughout the local GP community via education sessions and GP letters. Patients were indirectly included in the process through the media campaign associated with the project and directly included via a letter from TTH to allow them to determine if they considered they still required the specialist appointment.

### Previous strategies

Our first attempt to address long wait referrals was not successful. In this first attempt, a list of long wait patients was sent to the referring GP to confirm if the referral was still current. This process proved time consuming for GPs and practice managers due to difficulty of data retrieval within practices and transiency of the patient and GP populations. Further, this additional workload was contrary to our aim of implementing a change process with minimal impact on current work practices.

### Rationale for the Minimum Data Set

Overwhelming negative feedback from hospital staff regarding referral content prompted the GP Liaison Officer to conduct an internal audit on consecutive new orthopedic referrals over a three month timeframe and found only 25% contained complete and appropriate information to adequately triage. Findings from an audit of existing referrals showed many referrals contained clinical information that was up to eight years old. Information regarding the duration of referral validity is limited, but it is suggested in the Australian Medicare Benefits Schedule (Note G6.1) to be 12 months. Poor quality referrals present a dual risk management issue. If high risk patients are ranked low on the triage scale, negative outcomes may result before the appointment date [6]. Conversely, if low risk patients are seen urgently, it may inappropriately delay high risk patients. One solution is to improve the adequacy of the information contained in the referral to enhance appropriate triaging [2,3]. Therefore, we developed a referral template containing a minimum data set for each of the specialties. The minimum data set was used to update clinical information for the long wait process and to ensure all appropriate investigations had been performed.

### Development of the minimum data set

To develop the minimum data set, we searched the grey literature and found numerous resources had already been developed in Australia [10-12]. Using these resources, and input from the relevant specialist all information potentially required for a referral was drafted into a template. This draft was refined at a subsequent meeting to ensure it met triage requirements for the specialist and was of acceptable length for GPs to complete in a standard consultation. Further refinement continued using a Delphi process, where the draft was emailed between the two groups until a consensus was reached. We have now developed templates for most specialist clinics at The Townsville Hospital. The templates are also available as a direct electronic referral document for registered GPs from their desktop to The Townsville Hospital via Townsville GP Network.

### Process of the "long wait" referrals

**Figure 1:**
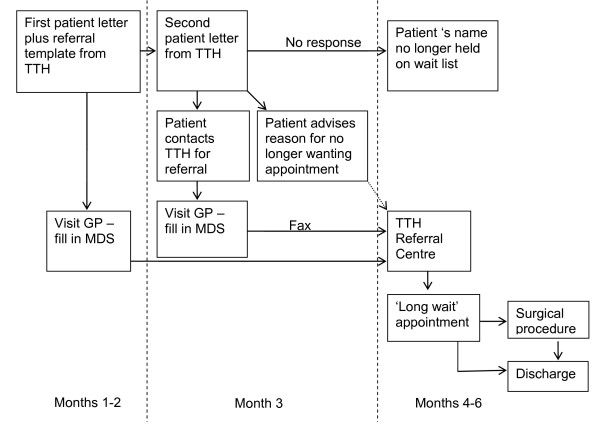
**Diagrammatic representation of the newly developed process for patients on the 'long wait' list to access a public hospital specialist appointment**.

The process to provide an appointment for long wait patients in Category 2 and 3 has evolved over time, and continues to evolve. The current process is shown in Figure 1. Patients on the long wait list are sent a letter directly from The Townsville Hospital clearly advising two options. Option A advises patients to 'Take no action' if the referral is no longer required and option B advises them to update the referral by presenting the referral template (included in the patient letter) to their GP within three months. Patients are advised in this letter that their name will no longer remain on the wait list if they choose Option A. If no response, patients are sent a reminder letter after two months. The three month timeframe was chosen on the basis of the standard timeframe for review of chronic conditions and to avoid an increased work load for GPs. The GP then returns the referral to The Townsville Hospital Referral Centre so that an appointment can be arranged. All patients whose referrals were returned in the long wait process were given an appointment.

Evening information and education sessions by relevant specialists were held at regular intervals to advise local GPs of the new referral template and minimum data set. They are also provided information on conservative management strategies by appropriate allied health professionals (e.g. senior musculoskeletal physiotherapist). Additional awareness strategies included presentations, education sessions and newsletters to practice managers and practice nurses. Importantly, the general community was informed of the process via local print, TV and radio. GPs were provided with a copy of the referral template prior to the first mail out to patients and a downloadable version was available on the Townsville GP Network website. A good working relationship and an open communication policy with The Townsville Hospital Referral Centre staff were essential to the success of this process.

## What happened

The first trial of the long wait process was conducted in 2008. In August of that year, 872 long wait orthopaedic patients were sent letters asking them to update their clinical information if they still wanted the appointment. A total of 101 patients responded, all of whom were seen at specially arranged clinics. This resulted in 16 procedures, confirming our belief there were patients on the long wait list in need of procedures. They had little likelihood of being seen under the previous process.

In 2009, letters were again sent to orthopaedic long wait patients (562) and also patients on the long wait list for ENT (1095), neurosurgery (544), urology (699), and general surgery (1241). A total of 532 patients updated their clinical information as a result of these letters. Again, all of these patients were seen at specially arranged clinics. The numbers of surgical procedures resulting from these appointments were: ENT 16; neurosurgery 1; orthopaedics 14; urology 8 and 138 for general surgery. At the end of 2009 the wait time for orthopaedics, ENT, neurosurgery, and urology was 2 years, and the wait time for general surgery was down to 1 year. The higher number of procedures in general surgery is explained by the shortened wait time to one year as only 30 of the 138 general surgery procedures were from the long wait timeframe.

In 2010, we are conducting the long wait process on the specialties of orthopaedics, ENT, neurosurgery, urology, general surgery, and for the first time vascular surgery and ophthalmology. By the end of 2010 we expect the wait time to be one year for general surgery, 18 months for orthopaedics and ENT, neurosurgery and urology, and 2 years for ophthalmology and vascular surgery. Since 2008 to date, a total of 6885 letters have been sent to long wait patients, 633 patients have responded by updating their clinical information and of those, 197 have required a procedure.

## Learnings

Since 2008, we have learnt the long wait process is achievable within the routine running of the surgical clinics, demonstrating it can co-exist with normal referral centre workflow. This process is cost effective in identifying the small number of people on the long wait list in need of a procedure. However, it is not cost neutral because of the extra resources required to hold additional clinics to process responding patients. It is anticipated that costs will reduce over subsequent years as the number of patients being sent letters also reduces.

There has been a positive change in attitude amongst staff involved in the long wait process regarding the issues of GP referral. This has come about by focussing on the clinical context of the referral using the minimum data set rather than focussing on the administrative process of the referral. All stakeholders benefit: GPs have access to consultant opinion for their patients; specialists have improved referral data enabling clinical management decisions at the first consultation; and patients who need procedures receive them. The success of this process and the resulting positive attitude to change forms a solid base for further reform.

In hindsight, this process would have been much easier to achieve if referral communication was electronic rather than paper based. An additional advantage of an electronic system would be the accuracy of referral data to accurately evaluate the process. The establishment of this process relied heavily on the inter-professional relationship between the GP Liaison Officer and the individual hospital specialists and hospital staff, which may be overcome by the use of incentives. This GP referral process has provided an equitable system for non-urgent 'long wait' patients to access a public hospital specialist clinic appointment and subsequent procedure if required.

## Competing interests

IS holds a small number of shares in Ramsay Health Care (ASX: RHC) which is an operator of private hospitals. Indirectly, the reduction of waiting lists in public hospitals could lead to reduced income for private hospitals. The authors LS, TP, AJ and GT declare that they have no competing interests.

## Authors' contributions

LS conceived the initial concept. LS and AJ participated in its design and coordination and led in its implementation. TP conducted the literature review and wrote the first draft and LS substantially contributed to the redrafts. AJ and IS reviewed and provided inputs to the drafts, contributed to the literature review. GT participated in implementation and with data collection. All authors read and approved the final manuscript

## Pre-publication history

The pre-publication history for this paper can be accessed here:

http://www.biomedcentral.com/1472-6963/10/303/prepub
